# A comprehensive review on diabetic foot ulcer addressing vascular insufficiency, impaired immune response, and delayed wound healing mechanisms

**DOI:** 10.3389/fphar.2025.1622055

**Published:** 2025-11-12

**Authors:** Chavan Aditya, Sarad Pawar Naik Bukke, Kuttiappan Anitha, Pandhare Meeraraje, Narayana Goruntla, Tadele Mekuriya Yadesa, Hope Onohuean

**Affiliations:** 1 Department of Pharmacology, School of Pharmacy and Technology Management, SVKM’s Narsee Monjee Institute of Management Studies Deemed to be University, Shirpur, Maharashtra, India; 2 Department of Pharmaceutics and Pharmaceutical Technology, Kampala International University, Kampala, Uganda; 3 Department of Clinical Pharmacy and Pharmacy Practice, Kampala International University, Kampala, Uganda; 4 Biopharmaceutic Unit, Department of Pharmacology and Toxicology, Kampala International University, Kampala, Uganda; 5 Biomolecules, Metagenomics, Endocrine and Tropical Disease Research Group (BMETDREG), Kampala International University, Ishaka-Bushenyi, Uganda

**Keywords:** diabetic foot ulcer, immunological responses, macrovascular contamination, microvascular, neuropathy

## Abstract

Diabetic foot ulcers (DFUs) continue to represent one of the most significant and costly complications related to diabetes mellitus, posing serious challenges to healthcare systems and resulting in considerable morbidity rates. This narrative review explores the complex pathophysiology of DFUs, focusing on the interplay between peripheral neuropathy, vascular insufficiency, and a weakened immune response, all of which contribute to delayed wound healing. Neuropathy leads to a loss of protective sensation, causing unnoticed repetitive injuries, while both microvascular and macrovascular complications reduce tissue perfusion and hinder angiogenesis. Additionally, immune dysfunction and exaggerated inflammatory responses raise the occurrence of infection and negatively affect the healing process. The clinical manifestation, progression, and key risk factors of DFUs were discussed in this review, emphasizing the importance of early detection, careful foot care, and routine screening in individuals who are at risk. Numerous therapeutic approaches are reviewed, including wound debridement, sophisticated wound dressings, offloading techniques, glycemic control, and adjuvant therapies such as growth factor administration, hyperbaric oxygen therapy, and negative pressure wound therapy. For optimal results, a multidisciplinary team combining of vascular surgeons, podiatrists, endocrinologists, and wound care specialists was included. The analysis also points out that promising advancements in bioengineered skin substitutes, intelligent dressings, and regenerative medicine hold promise for the treatment of DFU in the future. Self-monitoring, appropriate footwear, and patient education are all important components of prevention, which remains a fundamental strategy. In the clinical management of DFUs, this narrative review incorporates the most recent research and highlights the value of proactive, customized, and multidisciplinary approaches.

## Graphical Abstract

**Figure:**
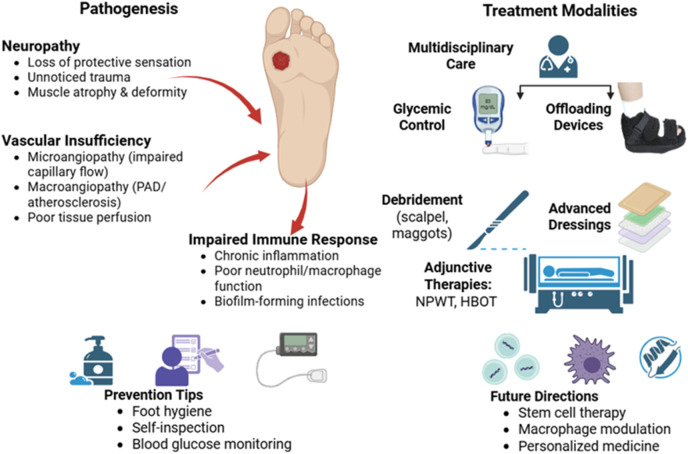

## 1 Introduction

Diabetes mellitus (DM), which has a high prevalence rate worldwide, has grown to be a serious concern. The two main kinds of this chronic illness are DM1 and DM2. A sizable portion of the global population is impacted by DM1, which primarily affects children and young people. Dm2 primarily affects adults and is frequently brought on by poor lifestyle choices, obesity, hormone imbalances, or a family history of diabetes. Diabetes can have far-reaching consequences, including the development of foot ulcers and other abnormalities in the body (Carstensen et al., 2020; Jiang et al., 2020; Barron et al., 2020; Ahlqvist et al., 2020). A difficult and confusing problem for the diabetic population is foot ulcers, a terrible and long-lasting consequence of untreated diabetes. If foot ulcers are not identified or treated, lower limb amputations may result. There are many different painful and upsetting ways that these ulcers can appear, and the general term for all of the painful varieties is neuropathic ulcers. Even if the underlying causes may vary, the main factor that causes severe suffering for those who have diabetes is systemic neuropathy, which is graphically caused by the disease’s unrelenting consequences (Allison and Flanagin, 2020; Afonso et al., 2021; Matheson et al., 2021). One of diabetes’s most common side effects, foot ulcers can be caused by a number of factors. People with diabetes usually develop neuropathy 10 years after the commencement of the disease, and lower limb amputation is frequently regarded as a possible consequence 20 years after the diagnosis. For those with type 1 diabetes, the risk of developing foot ulcers is 5%–10%, whereas DFU are inevitable for about 15% of people with type 2 diabetes. The fact that diabetes can lead to a variety of issues that affect various body organs must be kept in mind (Calcutt, 2020; Gibbons, 2020; Sasaki et al., 2020). People who have diabetes-related foot ulcers may experience severe distress and difficulty due to the common consequence of ankle joint ulcers. These ulcers, which first appear as calluses, are mostly found beneath the foot’s plantar surface. In the absence of prompt attention and appropriate care, these calluses progressively worsen and develop into serious ulcerations. DFU account for most amputations among diabetics, and the risk of lower limb amputation is notably elevated at 5 to 10 percent within 20 years of the onset of diabetes (Chamberlain et al., 2022; Edmonds et al., 2021; McDermott et al., 2023). A comprehensive evaluation of the literature on diabetic foot infections (DFIs) and ulcers is required in light of the aforementioned information. This review aims to explore the intricate biology of foot ulcers, the wide range of symptoms, the epidemiological consequences, and the important aspect of classification in this field. A thorough investigation of pharmaceutical research and DFU treatment choices can provide insight into various medications and therapeutic approaches that can promote wound healing and successfully eradicate infection (Ho et al., 2013; Wang et al., 2023).

## 2 Pathophysiology of DFU

**FIGURE 1:**
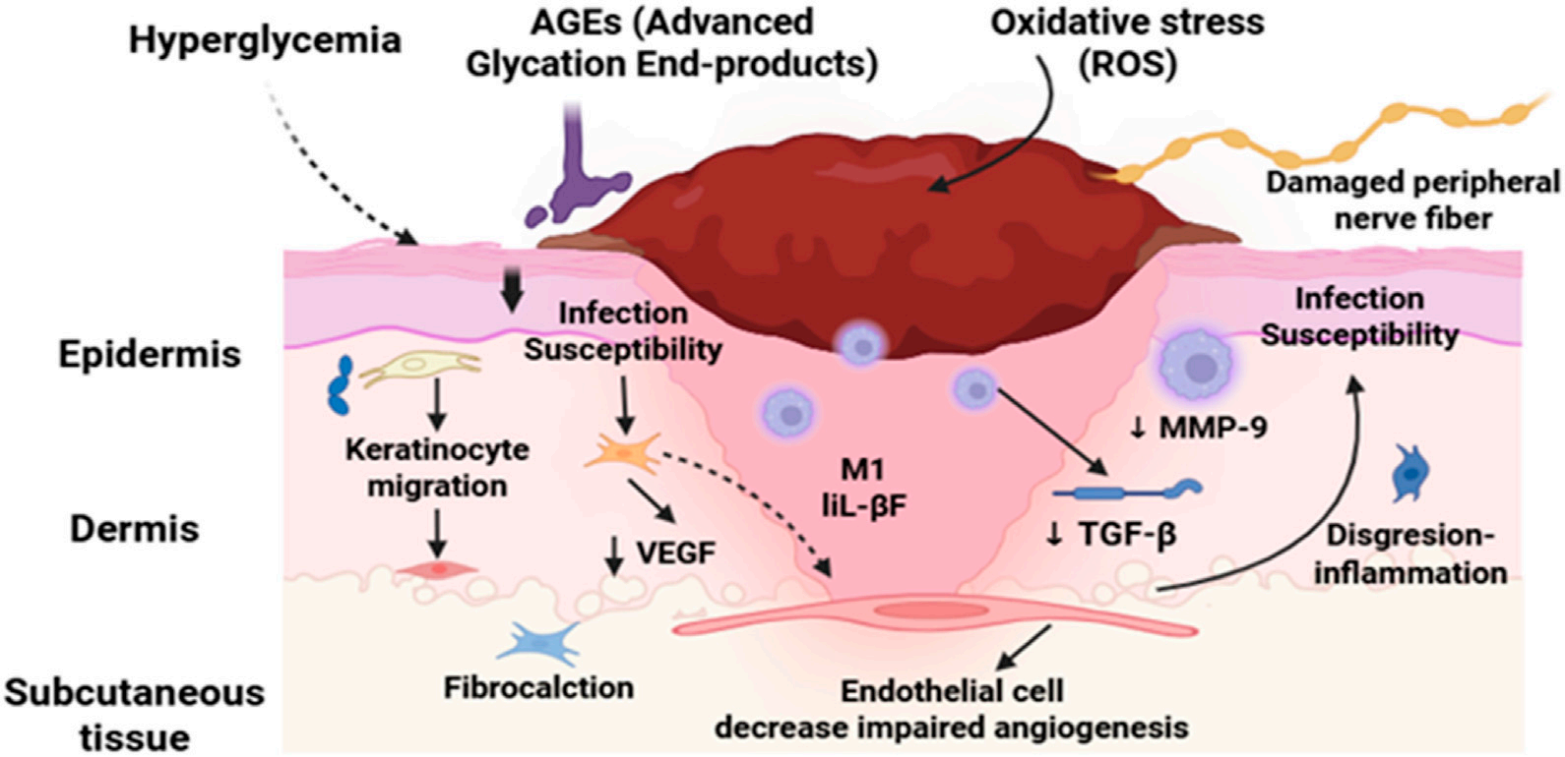
Pathophysiological mechanisms of DFU.

A sobering reminder of the severity of its effects is provided by the fact that diabetes is the primary cause of more than 60% of lower extremity amputations that take place globally without trauma (Creager et al., 2021; Lopez-De-andres et al., 2022; Mohammed et al., 2022; Saaiq, 2023). DFUs endanger patients’ quality of life in addition to causing significant financial impact. Due to the high risk of infection and the potential for limb amputation, DFUs are a painful and dangerous condition that requires immediate medical attention (Hashempour et al., 2024; Jodheea-Jutton et al., 2022; Gupta et al., 2021). There is a striking global impact of DFUs. Since it is estimated that a DFU occurs globally every 20 s, effective management methods are vitally needed. These ulcers, which impede the body’s natural healing process, often occur in diabetics and worsen over time due to neuropathy or peripheral vasculopathy. In Figure 1, the non-healing nature of DFUs is further compounded by systemic issues and intrinsic characteristics. A matrix transforming growth factor beta (TGF), vascular endothelial growth factor (VEGF), and metalloproteinase-9 (mmp9) (Da Costa Oliveira et al., 2021; Lazzarini et al., 2024a; Thotad et al., 2023).

A better understanding of the pathophysiology of DFUs has shed light on the basic processes responsible for impaired healing. Delays in DFU healing may be caused by a number of factors, including reduced neurotrophic support, neuropeptide production, and inadequate cutaneous blood flow as a result of microangiopathy. Abnormal scarring, excessive inflammation, poor keratinocyte migration, and poor mechanosensory adaptation can all hinder the healing process (Jethwa et al., 2024; Voza et al., 2024; Zheng et al., 2023). Despite the progress made in the treatment of DFU, obstacles need to be addressed. Although cellular therapy, growth factor therapy, and bioengineered skin are available, DFU restoration remains difficult. This is especially true in cases when follicular stem cell deficits are detected, such as in dermal vascular endothelial cells (Boulton et al., 2024; Vas et al., 2020). To resolve these problems, a thorough understanding of DFU pathophysiology is required. By investigating the fundamental factors that contribute to the beginning and progression of DFUs, researchers and medical professionals might develop more effective treatment regimens. With a focus on novel therapeutic approaches that have promise for treating this incapacitating illness, this study investigates the pathophysiology of DFUs (Bereda, 2022; Deng et al., 2023; Raja et al., 2023). DFUs remain a major concern for diabetics and healthcare systems around the world, to sum up. The complex pathophysiology of DFUs must be understood in order to develop effective treatment plans. Research into novel therapeutic alternatives like cellular therapy, growth factors, and bioengineered skin can help doctors and researchers improve patient outcomes and lessen the burden of DFUs (Ho et al., 2013; Wang et al., 2023).

## 3 Clinical presentations, risk factors for DFU

**FIGURE 2:**
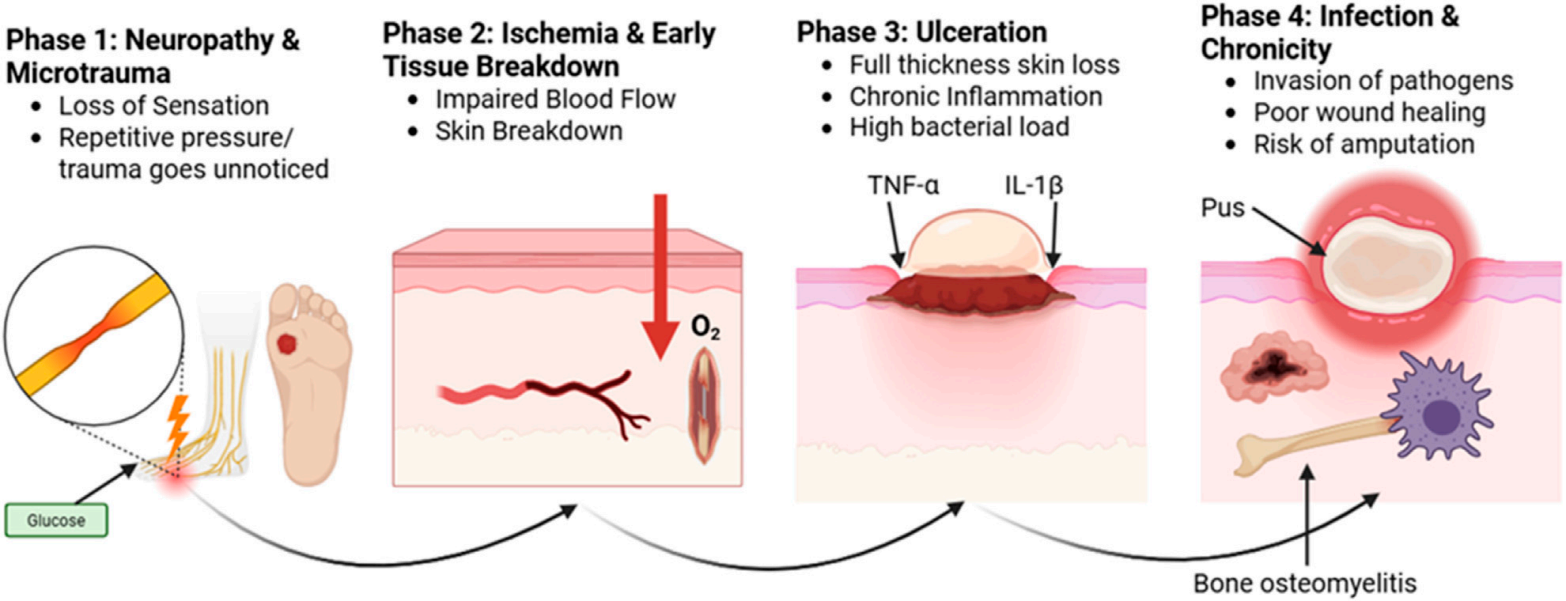
Phases of ulcer formation.

DFUs should be treated promptly and carefully in diabetic individuals. Although the ulcers are chronic in character and include an ischemia component, they are neuropathic in nature. The four stages of DFU are ulcer, callus, subcutaneous hemorrhage, and tumor necrosis factor alpha and interleukin 1 (Figure 2). When DFU issues occur, people’s quality of life is significantly diminished. Regular evaluation of diabetes is necessary to identify any potential problems early and treat them effectively. Callus, peripheral neuropathy, foot deformity, and an ulcer history are important risk factors that can be used to identify foot disorders early. The type of therapy and treatment that are needed will depend on the severity of the problem. An evaluation must include a thorough search for clinical presentations, physical examination results, and signs and symptoms of DFUs. Assessing the foot’s perfusion and feeling, as well as recording any changes in its color, temperature, or texture, are all part of this. Despite being fully aware of the risk factors that contribute to DFUs, both the general public and medical professionals frequently overlook the significance of performing foot exams before beginning diabetes treatment. Similarly, too often, neuropathic feet are disregarded until they cause discomfort, ulceration, or infection. In order to address this issue, a particular approach that prioritizes routine DFU risk factor screening and teaches diabetics the importance of foot care is necessary. Healthcare personnel can lower the occurrence of DFUs by using prevention and early intervention strategies. This will improve results and overall diabetic foot care. It is essential to give diabetes patients, clinical staff, and medical professionals a thorough understanding of the various risk factors that contribute to the development of DFU.

This include teaching people the value of having clean feet, how to choose and wear appropriate footwear, performing routine foot exams, and promptly reporting any abnormalities. With the right knowledge and abilities, patients and medical professionals can work together to prevent DFU and lessen their impact on those who already have the condition (Jayalakshmi et al., 2020; Kuang et al., 2021; Polikandrioti et al., 2020; Shah et al., 2020). DFU should be treated by an integrated group of medical specialists. This team may include physiotherapists, orthopaedic surgeons, vascular surgeons, endocrinologists, occupational therapists, social service workers, nurses, dietitians, and infectious disease specialists. Because of their complex needs, which often result from the interplay of multiple pathways, these patients require care coordination (Blanchette and Associates, 2020, Ayada et al., 2021; Brekelmans et al., 2023; Musuuza et al., 2020). Diabetes has serious side effects, including foot ulcers and amputations, which both significantly lower quality of life and increase death rates. Since a sizable portion of patients have risk factors, healthcare practitioners may be able to prevent these issues by implementing interventions in care pathways. In order to improve the management of diabetes and related complications, including foot ulcers, team-based care has been suggested as a component of a coordinated strategy (Nehad et al., 2024).

## 4 Multidisciplinary treatment approaches

**FIGURE 3:**
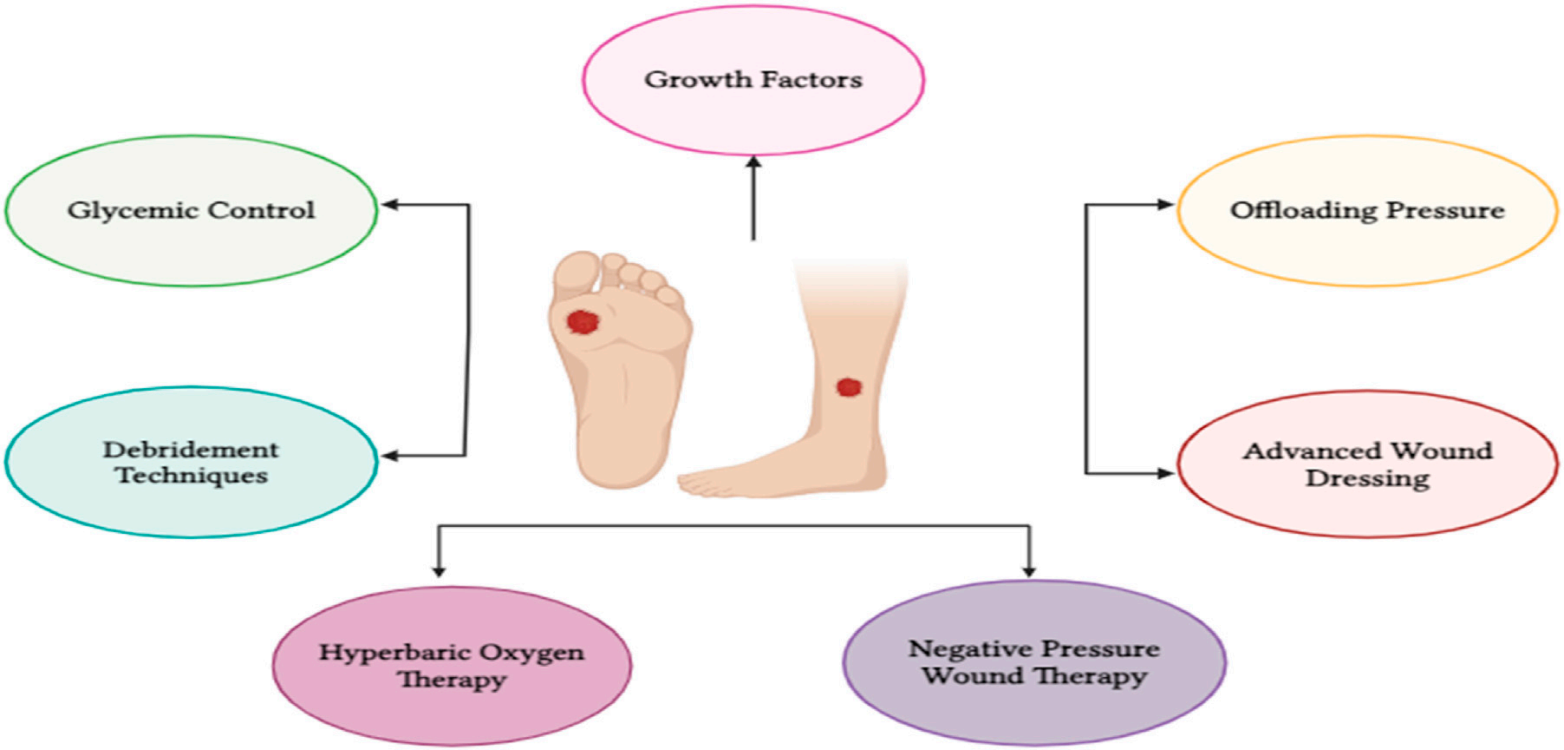
Treatment approaches for DFU.

There are various treatment approaches and adjuvant therapies are available for DFU that are glycemic control, offloading pressure, advanced wound dressing, debridement techniques, hyperbaric oxygen therapy, negative pressure wound therapy, growth factors (Figure 3). These techniques are thoroughly described below.

### 4.1 Glycemic control

In order to effectively treat and prevent DFUs, optimal glycemic control is essential. It is well known that hyperglycemia significantly affects keratinocyte migration, fibroblast proliferation, and neo angiogenesis, among other aspects of wound healing. Maintaining a hemoglobin A1C level below 6.9%, which is close to normal, can reduce the risk of developing a foot ulcer in diabetics by around 50% when compared to those whose A1C level is higher than 7.5%. The severity and duration of hyperglycemia are substantially associated with the likelihood of developing diabetes complications, as demonstrated by the Diabetes Control Complications Trial (DCCT) and the United Kingdom Prospective Diabetes Study (UKPDS). It is wise to start early and step up treatment efforts to get blood sugar levels as close to the normal range as possible (Standards of Medical Care in Diabetes, 2006; Diabetologii and Wewnętrznych, 2020). The ideal range for glycemic levels is 4.05–6.05 mmol/L, or 70–110 mg/dL. Maintaining normal cholesterol levels is essential, with a low-density lipoprotein concentration of less than 2.59 mmol/L, or 100 mg/dL, being the ideal target. A blood pressure reading of 130/80 mmHg should be the target. It has been shown that hyperlipidemia and hypertension worsen atherogenesis in diabetics with compromised endothelial function, which makes this all-encompassing approach to glycemic control crucial. By taking a systematic and thorough approach to these concerns, people with diabetes can significantly reduce their chance of developing DFUs and other associated problems. Their quality of life and general health will improve as a result. For patients and medical professionals alike, it is essential to stress the importance of careful and regular glycemic control. This can be accomplished by putting in place a multimodal strategy that involves regular exercise, lifestyle modifications, appropriate pharmaceutical administration, and continuous key marker monitoring. People with diabetes can live longer, healthier lives by reducing the burden of DFUs and associated issues with a proactive, tailored approach (Mat Saad et al., 2013; Sabapathy and Madhu, 2016).

According to Danesh and colleagues (2024), the average age group for the study is between 60 and 65. Research revealed a significant association between a person’s previous experience of DFU and the likelihood of developing the condition again. Of the patients in the control group, 43.1%, 50.7%, and 6.2% had good, moderate, and poor control serum HbA1C levels, respectively, while patients in the case group had moderate (7%–10%) and poor control (10%–13%) levels, at 78.5% and 12.3%, respectively (Danesh et al., 2024). A study conducted in 2023 by Dutta et al. identified the way that glycemic control influences DFU healing. A prospective nested cohort study was carried out on patients with poorly managed diabetes (glycated hemoglobin [HbA1c] >9%) with neuropathic DFU that lasted longer than 2 weeks. All of the patients received standard diabetes and ulcer treatment for a duration of 12 weeks. The cohort was divided into groups according to the healing status of the ulcer. We compared the ulcer area and glucose levels at 4 and 12 weeks of follow-up. During the first 4 weeks of treatment, early and rigorous glycemic control is associated with greater DFU healing, irrespective of the original ulcer area (Dutta et al., 2023).

### 4.2 Offloading pressure

Diabetes mellitus long-term effects of neuropathy and/or vascular damage have been associated to an elevated risk of foot ulcers in approximately 10% of diabetics. Infection and other serious side effects from the sores might result in hospitalization and, in 20% of cases, amputation. The application of best practices, such as debridement, unloading, moist wound healing, giving antibiotics for osteomyelitis, and teaching patients how to wear appropriate footwear, has been shown to greatly enhance clinical results when it comes to treating foot ulcers (Armstrong et al., 2023; Ouyang et al., 2021). It is crucial to stress how important it is for healthcare professionals to receive thorough training on these best practices. Ensuring that medical personnel receive training on the most effective treatment techniques may help us reduce the incidence of complications and improve overall treatment standards for patients with DFUs (Pourkazemi et al., 2020; Tuglo et al., 2022). The study of innovative wound care treatments for DFUs, including negative pressure wound therapy, improved wound dressings, and bioengineered skin substitutes, has exploded in the last 10 years. Applying these novel high-tech therapy modalities has produced promising initial outcomes. Physicians can more easily monitor the healing process of foot ulcers and choose the best course of treatment when they use high-resolution digital imaging. (Rayman et al., 2020; Swaminathan et al., 2024). For DFU, unloading should be a part of the healing process in order to avoid problems. Non-traumatic lower limb amputations are caused by DFU in around half of cases. Diabetes raises the risk of lower limb amputation and DFUs considerably. This has been shown to help individuals with DFUs heal, according to many meta-analyses. These therapies often involve additional basic care practices in addition to appropriately fitting footwear and providing foot support. Using casting procedures, a wide variety of foot ulcers are treated. Although the most evaluated offloading methods were casting processes, the most recent published meta-analysis revealed that bespoke footwear was the most effective offloading technique (Jones et al., 2024; Lazzarini et al., 2020; Luo et al., 2022). Although numerous studies and randomized trials comparing various offloading strategies have been conducted, the models used to assess the effects of footwear in conjunction with offloading insoles and greater depth remain inconsistent. Numerous challenges arise when pressure unloading is applied to the DFU, and methodological and theoretical issues must be properly addressed in any investigation. Investigating and utilizing current technologies is crucial to resolving these problems and improving the effectiveness of unloading therapy (Blakely, 2016; Hemler et al., 2023).

According to Lazzarini et al., non-removable offloading devices may increase the number of new lesions while also encouraging infection control, cost-effectiveness, and adherence. They might also lead to a decrease in infections and plantar pressure, although they might also cause more new transfer lesions. Offloading devices likely stretch the Achilles tendon and promote longer-lasting healing when compared to devices alone, but they also likely increase the risk of developing new heel ulcers (Lazzarini et al., 2024b). Hemler and others Diabetics who have difficulty reducing excessive plantar pressures (PPs), which remain a major risk factor, are more likely to develop lower limb ulcers and amputations. The footwear is designed to mimic a typical shoe that patients would wear every day in an attempt to improve adherence. Evaluations of the footwear’s initial pressure offloading and user perception in people without and with diabetes, respectively, yield encouraging results for its potential in the future. In conclusion, this smart footwear aims to prevent and treat DFUs and enhance patient usability, which will ultimately prevent lower limb amputations (Hemler et al., 2023).

### 4.3 Debridement techniques

Debridement refers to the proper removal of dead or diseased tissue from a wound. An important part of wound care is debridement, which promotes healing and prevents complications. The amount of the ulcer’s debridement depends on its size and depth. Reducing ulcers may also be necessary to release pressure in the affected area. Removal of some or all of the ligamentous, muscular, or bony structures that contributed to the ulcer’s development is part of this (Akkus and Sert, 2022; Dayya et al., 2022). Debridement is typically used to reduce ulcers in order to facilitate adequate cleansing and dead tissue evacuation. Debridement is necessary to treat any blood clots that may have formed during surgery and to remove any necrotic tissues that were obtained as a result of the process when treating the ulcer after surgery. An effective debridement treatment is required to promote wound healing and avoid infection (Moghaddam Ahmadi et al., 2024; Ning et al., 2023). Using sharp tools like scalpels or scissors, sharp debridement is frequently performed to remove dead tissue. This technique aids in debridement and flattening the edges of ulcers, which promotes healing overall. On the other hand, necrotic tissue can be removed while healthy tissue is preserved when proteolytic enzymes are used during enzymatic debridement. Bed sores, facial ulcers, and forearm ulcers respond well to this treatment; however, DFUs may not allow it to reach the deep necrotic tissues. The goal of DFU debridement treatments is to remove dead tissue to promote healing. They can be classified as either non-mechanical (using natural enzymes or dressings to gradually soften and remove damaged tissue) or mechanical (using surgical instruments, water jets, ultrasound, or even sterile maggots to physically clean the wound). Every technique aims to accelerate healing and avoid infection or additional issues, but each has advantages and disadvantages based on the extent of the wound and the patient’s general condition (Cazander et al., 2020; Dayya et al., 2022). Autolytic debridement breaks down necrotic tissue by using the body’s natural fluids and processes. Necrotic debris can extrude more easily because of the moist environment that dressings and ointments create inside the ulcer. Enzymes and the fluid’s moisture content assist autolytic debridement, which breaks down the tissue. Moist or dry dressings are used in mechanical debridement to remove necrotic tissue. The diseased region can be cleaned and stirred throughout this treatment with towels, gauze sponges, or Whirlpools (Nowak et al., 2022; Tran et al., 2023). Despite being a useful technique for removing necrotic tissue, mechanical debridement will cause pain and irritation to nearby healthy tissue, which will impede the healing process. It is possible to discard both necrotic and healthy tissue, which could be detrimental to the body’s natural ability to regenerate. The debridement technique used to treat ulcers and wounds should be closely evaluated in order to maximize healing results and minimize side effects (Ning et al., 2023; Baig et al., 2022).

Infection control is essential for improving the prognosis of DFU patients. DFIs are difficult to manage due to the absence of accurate indicators to measure microbiological activity. Frequently, the diagnosis is made only on the basis of clinical judgment. In the early stages of DFU, monomicrobial infections are common, while in the middle to late stages, polymicrobial infections are seen. Wound healing may be hampered or stopped by polymicrobial bacterial infections. Current clinical guidelines suggest that patients with DFI should get systemic antibiotic therapy. The severity of the disease typically dictates the type of antibiotic used; broad-spectrum empirical antibiotic therapy is used for mild-to-moderate infections, while antibiotics that target aerobic Gram-positive cocci are used for severe infections. The proper use of antibiotics is a major element in the prognosis of DFU. IDSA advised using narrow-spectrum antibiotics for as short time as possible and ceasing to use them as soon as the symptoms went away (Wang et al., 2022).

When treating difficult wounds, the patient’s general health must come first. Diabetes patients must pay close attention to their blood pressure, renal function, cholesterol, blood glucose control, dietary guidelines, and general health. People’s education regarding diabetes is a crucial part of its treatment. Frequent foot care helps prevent infections and aid in the healing of ulcers. This include applying moisturizer, donning suitable footwear, and performing regular checks. Issues like poor circulation and nerve damage require special attention. In severe cases, doctors could advise patients to consult specialists to improve blood flow. Use tools such as vibration tests and monofilaments to check for nerve damage. If neuropathy is found, it is essential to teach foot care and stop its progression. Ulcers usually appear on the top of the foot in those with poor circulation and on the bottom of the foot in those who have suffered nerve damage (Mat Saad et al., 2013).

The use of maggot therapy on a patient with a DFU that was unresponsive to treatment was identified by Rasouli et al. Great care was used when applying the maggots to the wound, and the healing process was closely monitored. Changes in the wound’s characteristics, such as increased granulation tissue development, decreased necrotic tissue, and improved healing process indicators, were seen in the study. Maggot therapy has several benefits when used to treat wounds. It may efficiently remove dead tissue, lessen the quantity of germs, promote the growth of new blood vessels, and enhance the healing process of wounds (Rasouli et al., 2024). Moghaddam et al. look into the features and results of DFUs that are treated with standardized wound care and surgical debridement. Silver spray, fibrinolysin ointment, and moist bandages were the main treatments used in the trial. In this study, about one-third of the patients required flap and graft secondary surgery. The recovery period was 24 days on average, although some patients were unable to recover because of underlying impairments. Long-term patient follow-up is necessary for future studies to evaluate quality of life and long-term therapy results (Moghaddam Ahmadi et al., 2024).

### 4.4 Advanced wound dressings

Sufficient wound dressings act as an extra outer layer that is applied or firmly affixed to the injured area. Traditional bandages like gauze and cotton wool are still widely used in healthcare facilities for acute wound therapy because they are inexpensive and easy to apply. To treat persistent non-healing wounds, a variety of advanced wound dressings based on bioactive polymeric materials and/or functional composites are being researched. These innovative bandages better control the healing process than traditional ones for chronic wounds, including DFUs. Advanced wound dressings are made by mixing polymeric materials with different active components. The dressings can be made to perform different tasks by adding biologically active substances that can trigger, inhibit, absorb, release, or respond to particular species (Alven et al., 2022; Jiang et al., 2023; Sathyaraj et al., 2023). To improve clinical performance, some sophisticated wound dressings may serve several purposes at once and promote various healing pathways. To better understand DFU, readers are provided with an overview of advanced wound dressings that employ bioactive polymers and/or functional composites. Advanced wound dressings have a variety of functions, including being barrier, antibacterial, hemostatic, active, stimulating, skin-regenerative, non-adherent, oxygen-releasing, and temperature-sensitive, depending on its mode of action. There are more subcategories into which these functions fall. Examples of antimicrobial wound dressings include wound dressings based on nanotechnology, photodynamic therapy, negative pressure wound therapy, and topical antimicrobial medications. In addition to film dressings, waterproof dressings, semi-permeable dressings, vapor-permeable dressings, hydrogel dressings, and absorbent dressings (such hydrocolloid, hydrophilic foam, and alginate dressings), barrier wound dressings are a highly fundamental and significant category (Farahani and Shafiee, 2021; Tavakoli and Klar, 2020). The materials that are used to make wound dressings can be further divided into different groups, such as foam dressings, dressings made of electrospun mats, dressings made of shells, dressings made of silica or silicate, polymer-silica composite wound dressings, dressings made of metal or metal oxide, natural polymeric wound dressings, and synthetic polymeric wound dressings. Customized wound care treatments are possible since each material group has distinct qualities and benefits. Dressings made of hydrophilic foam provide cushioning, while hydrocolloid dressings absorb moisture. Metal composite dressings, such as silver dressings, also have antibacterial properties, while silicone dressings are flexible and adhere well (Farahani and Shafiee, 2021; Shi et al., 2020; Stoica et al., 2020). For the treatment of DFU, a comprehensive classification system for the most cutting-edge wound dressings now on the market is therefore recommended, based on material types and methods of action. By using this classification system, medical professionals would be better equipped to understand the wide range of wound dressings and choose the optimal one for their patients. By considering the unique requirements of each wound in addition to the intended treatment goal, physicians can improve patient outcomes and expedite the healing process (Ji et al., 2021; Monteiro-Soares et al., 2020a; Monteiro-Soares et al., 2020b). The advantages of creating improved wound dressings using functional composites and/or bioactive polymers are thoroughly examined, with an emphasis on how they could enhance patient outcomes and completely transform the management of DFU. Among the many benefits of advanced wound dressings are enhanced wound healing, decreased infection rates, better patient comfort, and cost effectiveness. They could potentially give patients more therapy options and satisfy unmet clinical needs in the repair of DFU. If current research and innovation in wound care continue to advance, it is expected that the use of functional composites and bioactive polymers will grow and aid in the treatment of DFU (Dumville et al., 2012; Lim and Jang, 2021).

When it comes to the healing process following a foot amputation in diabetics, Serrudo et al. emphasize the significance of appropriate dressings. They employed a spray application of silver sulfadiazine, lidocaine, and vitamin A soaked into gauze since recovery can be intricate and slow, particularly in high-risk individuals. This method accelerated and improved the wound’s healing, demonstrating how the appropriate dressing can significantly reduce the risk of additional amputations (Serrudo et al., 2024). Chen et al. recommend reevaluating past research with improved reporting guidelines. They examined the caliber of data supporting various wound care methods. In terms of their findings, the majority expressed little to no trust. Only two therapies showed moderate-quality evidence: sucrose-octasulfate and a patch composed of platelets, fibrin, and white blood cells. Negative pressure therapy, placenta-based products, topical oxygen, and hyperbaric oxygen were the four others with poor support quality. In general, there isn’t much evidence to support many of these techniques, and more well-planned clinical trials are required to determine what actually promotes wound healing (Chen et al., 2024).

## 5 Adjunctive therapies

In order to promote wound healing by creating a safe and effective environment, negative pressure wound therapy (NPWT) was selected as the treatment option. The healing process can be accelerated by autologous skin grafting after wound debridement. Various macromolecules, cellular components, frameworks, and even vascularized tissues can be found in skin substitutes made using tissue engineering techniques for chronic wounds. The effective treatment of chronic wounds has shown promise with these substitutes (Seidel et al., 2022; Tang et al., 2023). Both EGF and VEGF play a major role in wound healing. Diabetic rats have demonstrated their use in healing by accelerating cell migration, proliferation, angiogenesis, and collagen deposition. Papain-urea complex is the alternative technique; it efficiently absorbs inflammatory exudates and necrotic tissue while also promoting the formation of granulation tissue (Cheng et al., 2020; White et al., 2021). Topical antibiotics and enzymatic debridement have also been shown to work well together in DFUs to lower the bacterial burden and speed healing. Electrically stimulated footbeds have also been demonstrated to significantly enhance DFU recovery. Growth hormones, medications, and cells can be delivered to the wound site by covering footbeds with biodegradable hydrophilic polymers (Dayya et al., 2022; Rayman et al., 2020). As a possible treatment for DFU, hyperbaric oxygen therapy has been reviewed extensively. The potential advantages of ozone therapy, stem cell therapy, low-power laser therapy, and xenografts in promoting wound healing are also being investigated. Additional clinical studies are required to validate their efficacy and assess their performance in real-world wound healing scenarios (Sharma et al., 2021; Wenhui et al., 2021; Baig et al., 2022; Kumar et al., 2023).

### 5.1 Growth factors

Autoimmune disease is associated with DM1, a chronic illness marked by abnormal insulin production. In contrast, the primary causes of DM2 are insulin insufficiency and resistance. In addition to these distinctions, both forms of diabetes frequently present with inflammatory and metabolic problems. Of these adverse effects, recurrent DFUs are the most common and problematic, particularly in people with peripheral artery disease and polyneuropathy (Baig et al., 2022; Edmonds et al., 2021; Bekele et al., 2022). In order to tackle this issue, researchers looked at the use of topical autologous biological drugs. Platelet-Rich Plasma (PRP), Platelet-Derived Growth Factors (PVGF), and loaded Synthetic Biodegradable Microspheres (SBMS) make up these medications. These medications make use of growth factors and proteases that are present in the patient’s plasma or in specially made dressings (Anitua et al., 2022; Gupta et al., 2021).

Many studies in this area have demonstrated the effectiveness of vacuum-based devices for the non-surgical healing of DFU that do not heal. By directly applying biological agents based on plasma to the wound, these devices offer broad-spectrum therapy. According to the study, using these methods helps chronic DFU heal much faster. We performed comprehensive clinical and instrumental evaluations on 65 patients with persistent wounds. For 6 weeks, half of the patients underwent biweekly bouts of vacuum-assisted closure (VAC) using customized plasmatic biological dressings. The remaining patients, however, received regular care (Wang et al., 2021; Yang and Choy, 2024). Healing area reduction during therapy and the overall number of patients treated were the main study features. The statistical analysis based on the Mann-Whitney test unequivocally shows that wounds treated with VAC heal more quickly after 6 and 14 weeks. These outcomes show how effective the vacuum-assisted closure method is at healing DFU that are hard to heal. Our study emphasizes the significance of expanding the use of plasma-based biological systems and continuing to develop medical devices since diabetes is becoming more common and has serious consequences. The results of the study show the possibility for commercialization of independent systems utilizing these technologies. The effectiveness and accessibility of treatment options for chronic DFU need to be improved as we gain a better understanding of the problems that diabetes causes (Wang et al., 2023).

### 5.2 Negative pressure wound therapy

To speed up the healing process, NPWT devices continuously apply a predetermined sub-atmospheric pressure inside wound dressings. The treatment of acute wounds, persistent ulcers, and surgical wounds has showed promise with this new approach (Gomez et al., 2020; Naser et al., 2022). First introduced in the 1990s, NPWT uses an electric suction device in conjunction with adhesive semi-permeable polyurethane foams to control infection and seal chronic skin wounds. NPWT was initially discovered to be a useful supplement to surgery in the treatment of DFUs. First, it was observed in relation to medically induced tissue hypertrophy. The convenience and security of NPWT are two of its primary advantages. This therapy is quick and efficient, and the healthcare provider can administer it at the patient’s bedside without any training. Due to recent advancements in semi-permanent device technology, NPWT technology is now more widely used in standard clinical settings (Normandin et al., 2021).

It's crucial to remember that NPWT or other novel approaches should never take the place of basic surgical principles. These guidelines cover proper vascular supply to the wound site, adequate debridement, and infection prevention and treatment. When combined with these core surgical concepts, NPWT can assist medical professionals in helping patients attain the best possible results (González-Ruiz, 2018).

### 5.3 Hyperbaric oxygen therapy

Another therapeutic option for DFUs is hyperbaric oxygen therapy, or HBOT. A state-of-the-art therapy approach called HBOT has revolutionized the treatment of several illnesses. For this treatment, a hyperbaric chamber is used, which allows patients to breathe 100% oxygen at far higher atmospheric pressures than usual. For the treatment of a number of ailments, such as air embolism, crush damage, carbon monoxide intoxication, and necrotizing fasciitis, HBOT is a well-liked option due to its proven therapeutic benefits. Although HBOT’s ability to heal wounds was initially investigated in the 1970s, new research has revealed its powerful impact. According to numerous studies, HBOT is essential for promoting neovascularization, stimulating fibroblast and keratinocyte migration, promoting optimum collagen deposition, and reducing edema, blood viscosity, and inflammatory reactions. These physiological changes lead to faster and more efficient healing of chronic ulcers, particularly in individuals with DM (Canha and Soares, 2023; Oley et al., 2020). When treating DFU that are chronic and do not heal, the National Academy of Sciences and the Institute of Medicine strongly recommend using HBOT. HBOT has a systemic effect by raising oxygen levels throughout the body, in contrast to conventional techniques like wound debridement and silver dressing, which mainly target the local wound bed. This systemic strategy promotes numerous local and general physiological changes in addition to hastening wound healing. Significant increases in dermal and epidermal levels, together with noteworthy improvements in collagen synthesis and neovascularization, have been repeatedly observed in clinical observations. With both commercial and private facilities, the Hyperbaric Medical Unit of the prestigious Clinical Research Centre in Kuala Lumpur is the only and leading hyperbaric center in Malaysia. Because of the facility’s cutting-edge equipment and knowledge, medical professionals now have more treatment alternatives. This has made it easier to find new ways to treat DFUs (Shi et al., 2020; Swaminathan et al., 2024).

The efficacy of HBOT in the treatment of DFU was assessed in this study using a good scientific methodology. Initially, surgical debridement and dressings, such as gauze pads soaked in saline, were used to treat diabetic foot pressure ulcers (DFPUs). To ascertain the impact of HBOT, several physiological and biochemical markers were thoroughly evaluated, including spot urine albumin, serum creatinine, lipid profiles, white blood cell (WBC) count and differential counts, glucose and HbA1c, and precise oral quality (EOQ). In order to assess the improvement in patients’ wellbeing before and after HBOT, this study employed two quality-of-life assessment instruments: the SF-36 and the Malay WHOQOL-BREF. Following the course of treatment, it is anticipated that all measured parameters will exhibit significant improvements, demonstrating HBOT’s efficacy in controlling DFU (Sharma et al., 2021; Wenhui et al., 2021; Bajuri, 2017).

## 6 Preventive measures and patient education

The first step in preventing DFUs is keeping the feet clean every day. Patients are recommended to check their feet every day for any wounds, blisters, redness, or swelling. Although it might not seem like much, this procedure is a great method to identify issues before they become serious. This care includes keeping the skin hydrated (but not between the toes), carefully cutting the toenails, and avoiding barefoot walking. Educating patients about these behaviors empowers them to take charge and avoid wounds that may result in infection or even amputation (Armstrong et al., 2023; Edmonds et al., 2021; McDermott et al., 2023). One of the best strategies to keep feet safe from harm is to wear shoes that fit properly. A tiny stone or a tight shoe seam might cause a wound without the diabetic realizing, especially if they have nerve impairment. Instructing patients on how to choose shoes that are comfortable, cushioned, and free of pressure points is essential. In certain situations, offloading devices or bespoke orthotics are advised to relieve pressure from high-risk areas, lowering the likelihood that ulcers would develop in the first place (Armstrong et al., 2023; Rayman et al., 2020; L. Wang et al., 2022). Education for patients must extend beyond simple foot care. It is crucial to comprehend the risk factors, which include weak circulation and neuropathy. Patients should be trained to identify warning symptoms, such as numbness or persistent pain, on their own, but professionals can also use tools like vibration sensors or the monofilament test to screen for nerve injury. Wounds can be kept from becoming chronic or necessitating amputations by promptly referring patients to specialists (such as vascular surgeons) when problems like decreased blood flow are identified. A single clinic visit is not enough to provide effective prophylaxis; it is a continuous practice. Organized education programs that are adapted to the patient’s literacy level and lifestyle have been shown in studies to reduce hospitalizations and ulcers. Personalized therapy, computerized reminders, and group training can all help people remember to take care of their feet. The most significant difference in long-term results is that patients who are aware of their disease are more likely to follow their daily schedules and seek care early (Miranda et al., 2021; Untari et al., 2024).

## 7 Future directions in DFU management

DFUs are turning into a serious public health issue on a worldwide scale. People with diabetes have a much higher chance of developing DFU for unknown reasons. To avoid and effectively treat DSU, it is essential to understand the complex process that underlies diabetic problems. To gain a better understanding of the impaired wound healing in diabetes, a meticulously crafted controlled rat model was developed. With the use of this model, the pattern of inflammatory cytokine expression in the wound tissue could be thoroughly investigated, revealing the specifics of the healing process (Baig et al., 2022; Jalilian et al., 2020).

The prevention of DFU formation was demonstrated to be significantly impacted by DBSN treatment. Macrophage polarization in wound tissues was directly correlated with the DBSN mode of action, according to other studies. More specifically, the pro-healing effects of M2 macrophage invasion aided in the healing process. This suggests that DBSN has a lot of therapeutic promise for DFU patients (Ashe, 2020).

Not just slow-healing lesions DFUs are a serious global health issue that significantly affects patients and healthcare systems. DFU-related amputations are expected to triple by 2030 if current trends continue, with cases rising at an alarming rate of 9% annually. Fortunately, with proper care, a significant portion of DFUs can be prevented. Using pressure-relieving techniques (such special shoes or unloading devices), controlling blood sugar levels, and treating infections as soon as they arise are all examples of preventive strategies. Prevention includes preserving quality of life and preventing long-term incapacity in addition to avoiding injuries (Armstrong et al., 2023; McDermott et al., 2023). Recent scientific discoveries are giving rise to renewed optimism regarding better care for chronic wounds, including DFUs. The body’s own healing processes are currently being studied by researchers, who are paying particular attention to how immune cells like macrophages react to damage and how skin cells might be “reprogrammed” to mend more effectively. Future treatments include hydrogels that lessen harmful oxidative stress, medications that aim to control inflammation, and stem cell-based therapy. Clinical techniques like improved dressings, skin substitutes, and negative pressure wound care are already in use and continue to produce positive outcomes. More high-quality studies are still required despite these advancements, particularly ones that involve individuals with varying ulcer severity levels. This is essential to guarantee that therapies work for everyone, not only in certain situations. In the future, preventing serious consequences and allowing people with diabetes to have better lives will depend on combining early identification with an integrated care plan (Boulton et al., 2024; Wang et al., 2022).

DFUs are localized lesions or sores on the feet that can result from diabetes-related issues and secondary infections. Amputation surgery is often required for DFUs, which can occur in up to 25% of diabetics at some point in their lives (Koreyba et al., 2022). Neuropathy, inadequate blood flow (ischemia), and foot abnormalities are the primary causes of DFUs. Due to nerve loss, neuropathic ulcers typically don't hurt because the foot’s sense of pressure or irritation is gradually diminished. This loss of feeling can cause skin to deteriorate, develop calluses, and crack, which makes it easier for infections to enter. Ischemic ulcers, on the other hand, can hurt because the area doesn't lose feeling, but the healing process is slowed down by inadequate circulation. Neuroischemic ulcers, which are a combination of nerve and blood flow shortages, or traumatic ulcers, can also result from foot deformities that generate inappropriate pressure or trauma (Sanjeeviraj et al., 2023).

The first step in effectively treating DFUs is to identify and treat the underlying causes as soon as possible. Adequate blood flow is the most important factor in healing, and in certain situations, surgery is required to fix it. Additionally, the wound must be meticulously cleaned by debridement, which is the removal of any dead or contaminated tissue. Early administration of the appropriate medicines is essential if infection is evident. Above all, blood sugar control is crucial since unchecked glucose will significantly hinder or perhaps completely prevent the healing process (Balasubramanian et al., 2021; Hinchliffe et al., 2024; Houlind, 2020).

## 8 Conclusion

A major consequence of diabetes that has far-reaching effects on both patients and healthcare systems is DFUs. The combination of neuropathy, ischemia, and structural abnormalities, which all increase the risk of infection and skin deterioration, usually fuels the illness. Attenuating this load requires prevention. Early treatment, education, and routine foot care can greatly lower the risk of developing ulcers. DFUs can be delayed or avoided by wearing protective shoes, maintaining proper glycemic control, and routinely checking for neuropathy or impaired circulation. Notably, teaching patients to recognize the early warning signs of foot issues encourages them to seek medical attention as soon as possible to prevent the issue from becoming life-threatening. A coordinated, multidisciplinary approach is required to manage the financial, emotional, and physical strain of DFUs. In order to guarantee foot protection, all parties involved in foot health—caregivers, patients, and medical professionals—have a role to play. The most effective way to combat DFUs is still to educate patients and inculcate good care behaviors.

Innovations in wound care technologies hold great potential as current research continues to reshape the management of DFUs. Innovative medicines that modulate the immune response and promote tissue regeneration, such as stem cell therapies and bioengineered dressings, show promise. It will take more excellent clinical research to prove their efficacy in diverse patient groups. The cornerstones of therapy, however, continue to be established treatments such as debridement, pressure offloading, infection management, and blood sugar control. Prompt diagnosis, customized treatment plans, and consistent monitoring are ultimately the best ways to improve healing outcomes and avoid recurrence. It is more important than ever to implement these best practices in routine care settings due to the rising incidence of diabetes worldwide. Diabetes patients can maintain their independence and high quality of life by avoiding complications, employing evidence-based treatment, and receiving patient-centered education.
